# Antegrade bile duct stone removal with double guidewire deployment using a novel double-lumen dilator

**DOI:** 10.1055/a-2781-6117

**Published:** 2026-02-05

**Authors:** Takeshi Ogura, Kimi Bessho, Junichi Nakamura, Nga Nguyen Trong, Hiroki Nishikawa

**Affiliations:** 1 38588 Pancreatobiliary Advanced Medical Center, Osaka Medical and Pharmaceutical University Hospital, Osaka, Japan; 2 38588 Endoscopy Center, Osaka Medical and Pharmaceutical University Hospital, Osaka, Japan; 3 13010 2nd Department of Internal Medicine, Osaka Medical and Pharmaceutical University, Osaka, Japan; 4 Department of Gastroenterology, Trong Nam Cancer Hospital, Hanoi, Vietnam

Antegrade stone removal via endoscopic ultrasound-guided hepaticogastrostomy (EUS-HGS) has been reported as an alternative technique after failed endoscopic retrograde cholangiopancreatography (ERCP 1 2 3 ). During antegrade stone removal, the pushing force exerted by the balloon catheter across the papilla into the intestine is important because, compared with transpapillary stone removal, this force may be insufficient. In addition, if the common bile duct is angulated, the pushing force can deviate from the correct axis needed to advance the balloon catheter. To overcome this issue, double guidewire deployment might be helpful because the combined stiffness of the guidewires provides better support. A novel double-lumen dilator (Meissa, Japan Lifeline, Tokyo, Japan) has recently been developed ( Fig. 1 ). The device has a 2.3-Fr tip and a maximum diameter of 7.4 Fr. An additional 0.025-inch guidewire can be inserted through a side hole located 2 cm from the tip. We herein describe technical tips for antegrade stone removal using double guidewire deployment with a double-lumen dilator.

**Fig. 1:**
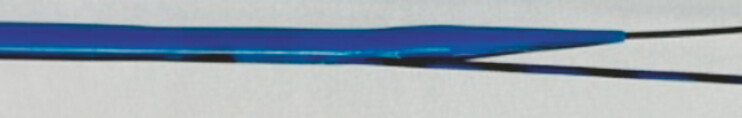
The novel double-lumen dilator (Meissa, Japan Lifeline, Tokyo, Japan).

A 77-year-old man was admitted to our hospital for the treatment of a common bile duct stone. He had previously undergone distal gastrectomy with Roux-en Y reconstruction for distal gastric cancer. Because enteroscopic-guided ERCP was unsuccessful, EUS-HGS was performed using a metal stent, with antegrade stone removal attempted 10 days later. First, an ERCP catheter was inserted into the common bile duct through the mesh of the EUS-HGS stent, and a 0.025-inch guidewire was deployed across the papilla ( Fig. 2 ). After removal of the metal stent using a forceps biopsy device through the working channel of the endoscope, the double-lumen dilator was inserted to dilate the papilla ( Fig. 3 ). Subsequently, an additional 0.025-inch guidewire was deployed through the side hole of the dilator ( Fig. 4 ). The pushing force is effectively transmitted owing to the increased stiffness provided by the double guidewires, and the common bile duct is straightened by double guidewires, and dilator was removed. And then, antegrade stone extraction using a balloon catheter was then easily and successfully performed ( Fig. 5 , Video 1 ). Finally, a plastic stent was placed without any adverse events.

**Fig. 2:**
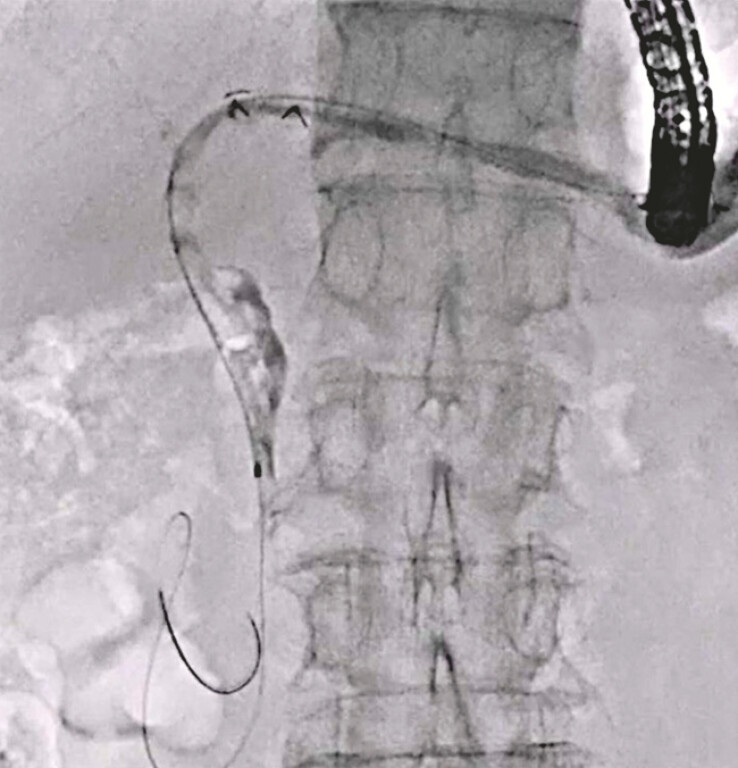
Guidewire deployment is attempted across the papilla.

**Fig. 3:**
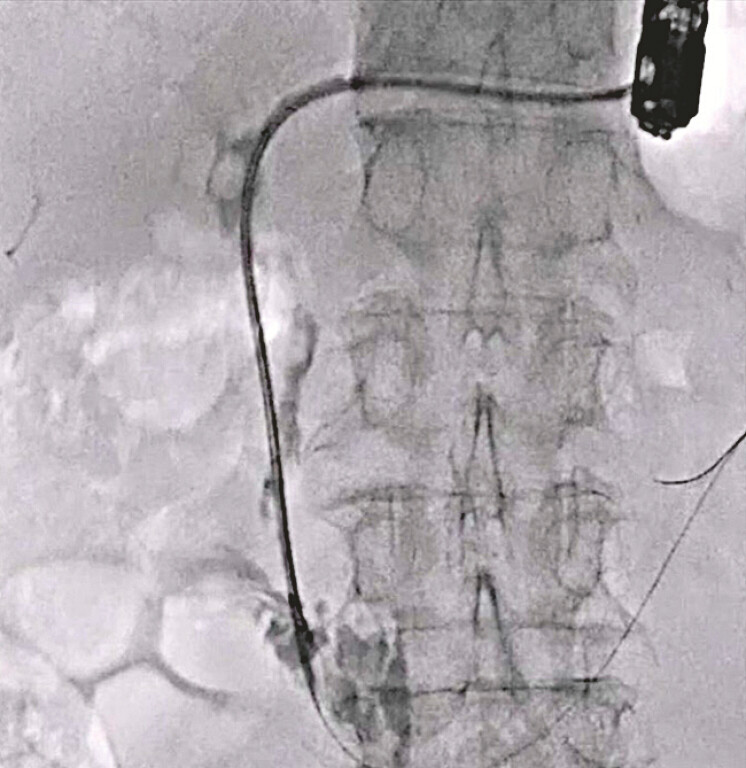
The ampulla of Vater is dilated using the novel double-lumen dilator.

**Fig. 4:**
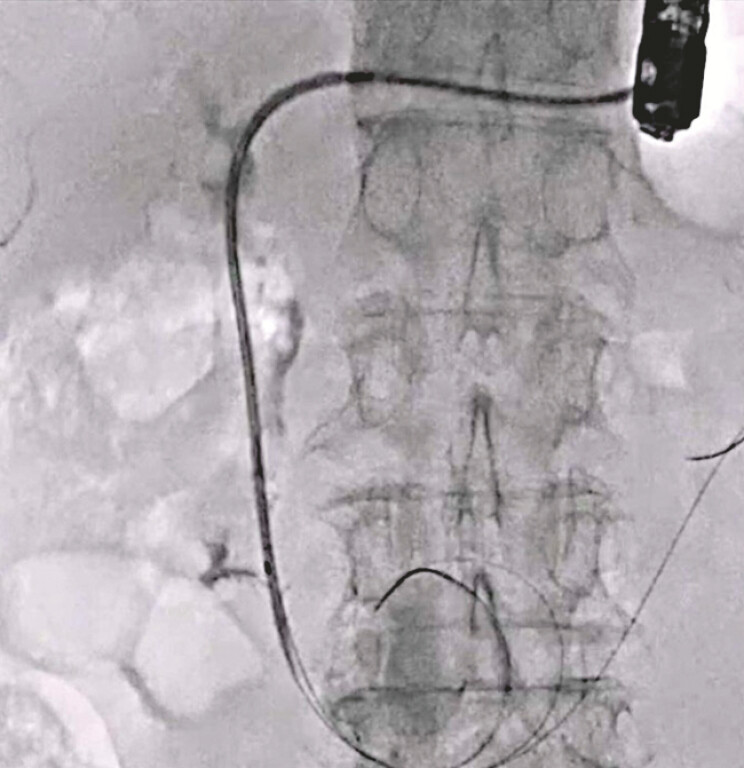
Additional guidewire deployment is performed.

**Fig. 5:**
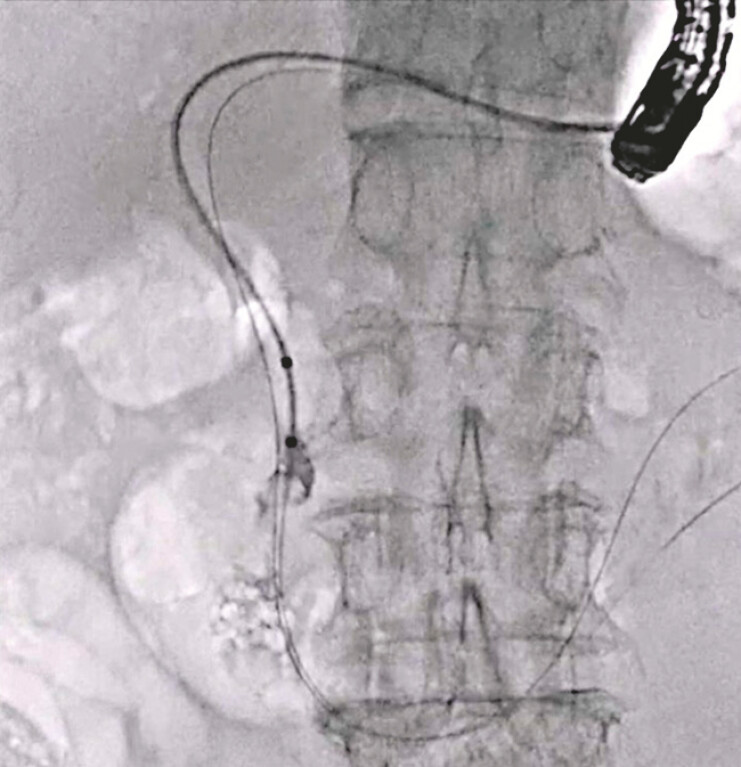
Stone extraction is easily performed using the double guidewire technique.

Antegrade stone extraction is performed under the double guidewire technique using the novel double-lumen dilator.Video 1

In conclusion, this novel dilator facilitates both the double guidewire technique and dilation of the papilla.

Endoscopy_UCTN_Code_CCL_1AF_2AF_3AB Endoscopy_UCTN_Code_TTT_1AR_2AH Endoscopy_UCTN_Code_TTT_1AS_2AH

## References

[1] Sato T Nakai Y Kogure H ERCP using balloon-assisted endoscopes versus EUS-guided treatment for common bile duct stones in Roux-en-Y gastrectomy Gastrointest Endosc 2024 99 193 203 10.1016/j.gie.2023.09.001 37709151

[2] Nagai K Mukai S Abe M Long-term outcomes after EUS-guided antegrade intervention for benign bilioenteric anastomotic stricture Gastrointest Endosc 2024 99 50 60 10.1016/j.gie.2023.07.052 37562548

[3] Ogura T Kawai J Nishiguchi K Transluminal intrahepatic bile duct stone removal using a novel spiral basket catheter via the endoscopic ultrasound-guided hepaticogastrostomy route (with video) Dig Endosc 2022 34 234 237 34459031 10.1111/den.14121

